# Machine learning algorithms for predicting mortality after coronary artery bypass grafting

**DOI:** 10.3389/fcvm.2022.977747

**Published:** 2022-08-24

**Authors:** Amirmohammad Khalaji, Amir Hossein Behnoush, Mana Jameie, Ali Sharifi, Ali Sheikhy, Aida Fallahzadeh, Saeed Sadeghian, Mina Pashang, Jamshid Bagheri, Seyed Hossein Ahmadi Tafti, Kaveh Hosseini

**Affiliations:** ^1^Tehran Heart Center, Cardiovascular Diseases Research Institute, Tehran University of Medical Sciences, Tehran, Iran; ^2^School of Medicine, Tehran University of Medical Sciences, Tehran, Iran; ^3^Cardiac Primary Prevention Research Center, Cardiovascular Diseases Research Institute, Tehran University of Medical Sciences, Tehran, Iran; ^4^Non-communicable Diseases Research Center, Endocrinology and Metabolism Population Sciences Institute, Tehran University of Medical Sciences, Tehran, Iran; ^5^Faculty of Electrical and Computer Engineering, Tarbiat Modares University, Tehran, Iran

**Keywords:** machine learning, feature selection, coronary artery bypass, prediction, mortality

## Abstract

**Background:**

As the era of big data analytics unfolds, machine learning (ML) might be a promising tool for predicting clinical outcomes. This study aimed to evaluate the predictive ability of ML models for estimating mortality after coronary artery bypass grafting (CABG).

**Materials and methods:**

Various baseline and follow-up features were obtained from the CABG data registry, established in 2005 at Tehran Heart Center. After selecting key variables using the random forest method, prediction models were developed using: Logistic Regression (LR), Support Vector Machine (SVM), Naïve Bayes (NB), K-Nearest Neighbors (KNN), Extreme Gradient Boosting (XGBoost), and Random Forest (RF) algorithms. Area Under the Curve (AUC) and other indices were used to assess the performance.

**Results:**

A total of 16,850 patients with isolated CABG (mean age: 67.34 ± 9.67 years) were included. Among them, 16,620 had one-year follow-up, from which 468 died. Eleven features were chosen to train the models. Total ventilation hours and left ventricular ejection fraction were by far the most predictive factors of mortality. All the models had AUC > 0.7 (acceptable performance) for 1-year mortality. Nonetheless, LR (AUC = 0.811) and XGBoost (AUC = 0.792) outperformed NB (AUC = 0.783), RF (AUC = 0.783), SVM (AUC = 0.738), and KNN (AUC = 0.715). The trend was similar for two-to-five-year mortality, with LR demonstrating the highest predictive ability.

**Conclusion:**

Various ML models showed acceptable performance for estimating CABG mortality, with LR illustrating the highest prediction performance. These models can help clinicians make decisions according to the risk of mortality in patients undergoing CABG.

## Introduction

Cardiovascular diseases -particularly coronary artery disease (CAD)-are the leading worldwide cause of death (1). Coronary artery bypass graft (CABG) surgery and angioplasty are the two primary revascularization methods used to treat CAD patients.

Several scores have been proposed for the assessment of cardiac operative risk, such as the European system for cardiac operative risk calculation (Euro-SCORE I and II) and the Society of Thoracic Surgeons (STS) (2–4). However, these scoring systems mainly evaluate in-hospital, and operative mortality and therefore are not generalizable to longer mortality. Many contributors, including previous medical comorbidities and procedural factors, are associated with higher mortality in patients undergoing CABG (5).

Machine learning (ML) models have been designed to predict outcomes in the cardiovascular medicine (6). These models have shown promising results compared to traditional risk scores with some advantages (7). In these artificial intelligence-based methods, the strongest predictors can be selected to train the system to predict outcomes using supervised learning. Afterward, the learning method is tested on unseen data for evaluation (8).

In light of this information, we aimed to use and compare different ML methods to predict one-to-five-year mortality after CABG.

## Materials and methods

### Study design and data collection

In this registry-based retrospective cohort study, baseline data from all adult patients (≥18 years old) in the Tehran Heart Center CABG databank enrolled from 2005 through 2015 were reviewed. The study was approved by the ethics committee of Tehran Heart Center (IR.TUMS.VCR.REC.1400.11.23). Due to the retrospective design of the study and data anonymization, the informed consent requirement was waived.

### Variables and outcomes

Various predictors were used for this study. Demographic variables included age, gender, and body mass index (BMI). Preoperative variables consisted of serum hemoglobin (Hb), high-density lipoprotein (HDL-C), low-density lipoprotein (LDL-C), total cholesterol, triglycerides (TG), creatinine (Cr), left ventricular ejection fraction (EF), diabetes, hypertension, opium consumption, smoking status, prior myocardial infarction (MI), preoperative heart failure (HF), and chronic obstructive pulmonary disease (COPD). The definitions were consistent with prior studies on this population (9). Perioperative variables, including cardiopulmonary pump utilization and ventilation hours, were assessed.

The primary outcome was mortality in one-year post-CABG. Secondary outcomes were two-, three-, four-, and five-year mortality.

### Data cleaning

At first, we omitted subjects if they had (1) any missing variable values (1,217 out of total 18,118), and or (2) implausible values such as: Hb < 5 or > 25, LDL-C > 400, TG < 20 or > 1200, HDL-C < 5 or > 100, and Cr < 0.2 or > 15. Excluding missing values was due to a sufficient sample number. For each endpoint during follow-ups, survivors with less follow-up duration than the cut point were excluded.

### Test/train split and feature selection

The study population was randomly assigned to the training cohort (70% of the patients) and the test cohort (30% of the sample) to validate the predictive models.

We ran a feature selection algorithm on the training data to select the most appropriate features. Top features were obtained from random forest model prediction in the train data using k-fold cross-validation (*k* = 5). In case of a strong correlation between two variables (confirmed by Pearson correlation r) that were also clinically related, the stronger predictor was used to set up the models.

### Oversampling and scaling

As one of the challenges in ML is imbalanced data, we used the synthetic minority oversampling technique (SMOTE) to balance our data in the training sample, which was performed after the test/train split as the data in the test sample should be unseen and remain unchanged. SMOTE technique creates synthetic data for the minority group, which was non-survivors in our study, to have an equal number of outcomes. The oversampling strategy, demonstrating the rate of the minority group to the majority, was tuned manually to have the best prediction model. Finally, a standard scaler was used to scale the data of each feature for developing prediction models. This standardizes features by removing the mean and scaling to unit variance. Oversampling and scaling were conducted for each follow-up data separately.

### Model development

To develop predictive models, six ML methods were utilized: (1) Logistic Regression (LR); (2) K-Nearest Neighbors (KNN); (3) Random Forest (RF); (4) Extreme Gradient Boosting (XGBoost); (5) Naïve Bias (NB) and (6) Support Vector Machine (SVM). All the models were designed using k-fold (*k* = 5) cross-validation. The parameters used for each model were tuned using the grid search method to increase the accuracy of the prediction. Each model was trained and tested for one-to-five-year mortality.

### Model performance evaluation

We evaluated the performance of ML methods using following indices. (1) Sensitivity; (2) specificity; (3) accuracy of prediction; and (4) area under the receiver operating characteristics curve score (ROC-AUC) by plotting true positive against false positive rate. As AUC is a measure of discrimination independent of threshold, we chose it as the major index to compare the performance of models. AUC was interpreted as follows. AUC ≥ 0.9, outstanding discrimination; 0.8 ≤ AUC < 0.9, excellent discrimination; 0.7 ≤ AUC < 0.8, acceptable/fair discrimination; 0.6 ≤ AUC < 0.7, poor discrimination; and AUC < 0.6, no discrimination (10). The threshold determines the cut-off to turn a projected probability into a class label which is normally set at 0.5 (50%). Finally, due to the highly imbalanced outcome and low mortality rate after CABG, the prediction threshold, which is usually set as 0.5, was tuned using k-fold (k = 5) cross-validation to adjust sensitivity and specificity.

### Statistical analysis

Baseline characteristics are represented as mean and standard deviation (SD) or percentage. Data were compared using the Pearson chi-squared test and Fisher’s exact test for categorical variables, and the independent sample t-test for continuous variables. Two-sided *p*-value < 0.05 was considered statistically significant. All statistical analyses and model development were performed using Python (3.10). LR, NB, RF, SVM, and KNN models were implemented using the scikit-learn (1.0.2) library (11), and XGBoost was developed using the XGBoost (1.6.0) Python library.

## Results

### Baseline and hospitalization characteristics

**FIGURE 1:**
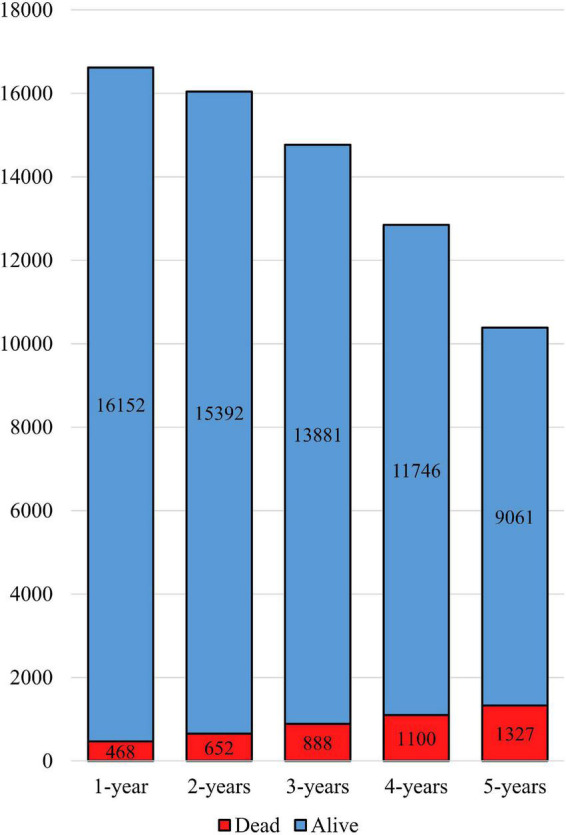
Number of survivors and non-survivors at each follow-up endpoint.

**FIGURE 2:**
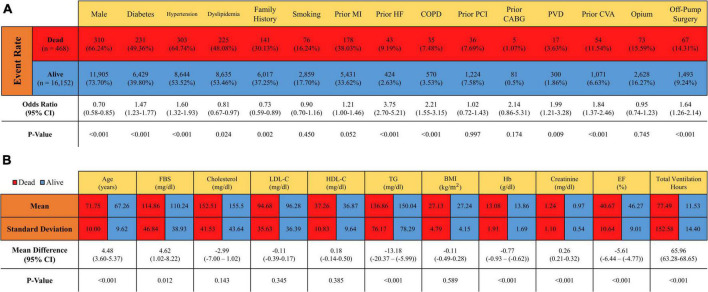
Comparison of baseline and hospitalization characteristics of survivors and non-survivors with one-year follow-up. MI, myocardial infarction; HF, heart failure; COPD, chronic obstructive pulmonary disease; CABG, coronary artery bypass grafting; PCI, primary cutaneous intervention; PVD, peripheral vascular disease; CVA, cerebrovascular accident; FBS, fasting blood glucose; LDL-C, low-density lipoprotein cholesterol; HDL-C, high-density lipoprotein cholesterol; TG, triglyceride; BMI, body mass index; Hb, hemoglobin; EF, ejection fraction.

The total cohort included 16,850 patients with isolated CABG (age: 67.34 ± 9.67, 73.51% male). Table 1 illustrates the baseline characteristics of the whole cohort. Among 16,620 patients with complete one-year follow-up, 2.81% (*n* = 468) died, followed by 4.06%, 6.01%, 8.56%, and 12.77% respective cumulative mortality rates at two, three, four, and five years of follow-up. Figure 1 indicates the number of survivors and non-survivors for each follow-up duration. Figure 2 compares the baseline characteristics between survivors and the deceased patients with a one-year follow-up. Non-survivors were more likely to be older and be afflicted with conventional CAD risk factors. Moreover, EF was significantly lower (40.67 ± 10.64 vs. 46.27 ± 9.01, *p*-value < 0.001), and ventilation hours higher (77.49 ± 152.58 vs. 11.53 ± 14.40) in non-survivors compared to the survivors.

**TABLE 1:** Baseline and hospitalization characteristics of the study cohort.

Variable	Total cohort (*n* = 16,850)
Age (years)	67.34 ± 9.67
Male	12,387 (73.51)
Hypertension	9,056 (53.75)
Diabetes	6,757 (40.10)
Dyslipidemia	8,970 (53.23)
Family history of cardiovascular disease	6,224 (36.94)
Smoking	2,989 (17.74)
Prior MI	5,681 (33.72)
Prior HF	475 (2.82)
COPD	610 (3.62)
Prior CABG	86 (0.51)
Prior PCI	1,277 (7.58)
PVD	319 (1.89)
CVA	1,155 (6.86)
Opium	2,755 (16.35)
Off pump surgery	1,592 (9.45)
BMI (kg/m^2^)	27.23 ± 4.17
FBS (mg/dl)	110.41 ± 39.23
EF (%)	46.11 ± 9.11
LDL-C (mg/dl)	96.20 ± 36.35
HDL-C (mg/dl)	36.86 ± 9.68
Cholesterol (mg/dl)	155.35 ± 43.55
TG (mg/dl)	149.68 ± 78.22
Creatinine (mg/dl)	0.98 ± 0.56
Hb (g/dl)	13.83 ± 1.70
Total ventilation hours	13.36 ± 31.02

Data are presented as mean ± S.D. or number (%); MI, myocardial infarction; HF, heart failure; COPD, chronic obstructive pulmonary disease; PCI, primary cutaneous intervention; PVD, peripheral vascular disease; CVA, cerebrovascular accident; FBS, fasting blood glucose; LDL-C, low-density lipoprotein cholesterol; HDL-C, high-density lipoprotein cholesterol; TG, triglyceride; BMI, body mass index; Hb, hemoglobin; EF, ejection fraction.

### Feature selection

**FIGURE 3:**
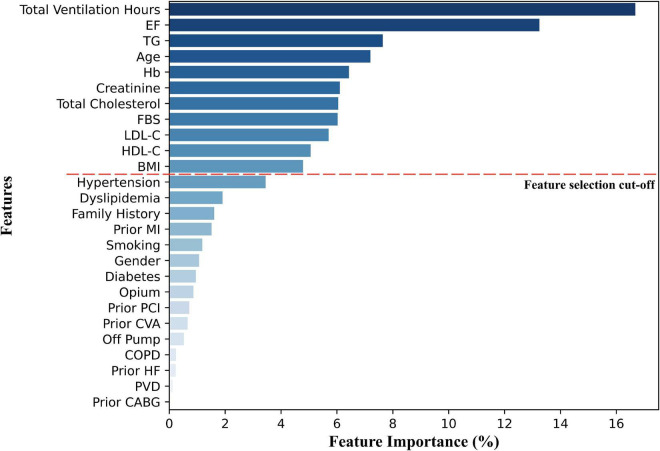
Feature importance based on the Random Forest model. MI, myocardial infarction; HF, heart failure; COPD, chronic obstructive pulmonary disease; CABG, coronary artery bypass grafting; PCI, primary cutaneous intervention; PVD, peripheral vascular disease; CVA, cerebrovascular accident; FBS, fasting blood glucose; LDL-C, low-density lipoprotein cholesterol; HDL-C, high-density lipoprotein cholesterol; TG, triglyceride; BMI, body mass index; Hb, hemoglobin; EF, ejection fraction.

We used the RF prediction model in the test data using k-fold cross-validation (*k* = 5) to rank all the features based on their importance. All correlations between the features were assessed by Pearson correlation r. Figure 3 demonstrates the features and their order for developing the models. Eleven features were chosen for predicting one-year mortality based on the RF model (Figure 3). Total ventilation hours and EF were the most predictive variables. Feature selection for secondary endpoints (longer follow-up mortalities) revealed the same predictor features with minor changes in their order.

### Model evaluation

Models ran for one-to-five-year mortality based on the top features selected and were evaluated on the test dataset. Table 2 compares the predictive values of different models concerning their AUC, accuracy, sensitivity, and specificity. All the models had at least acceptable performance for one-year mortality (10), with LR presenting the highest [AUC (95% CI) = 0.81 (0.77–0.85)] and KNN the lowest discriminatory ability [AUC (95% CI) = 0.72 (0.67–0.76)]. Moreover, all techniques illustrated acceptable performance (AUC > 0.7) for predicting two-to-five-year mortality (10), excluding the SVM model. After tuning for the threshold, the highest sensitivity was obtained in the LR model (72.99%), while the highest specificity and accuracy were calculated as 85.67% and 84.96% for the NB model, respectively.

**TABLE 2:** Evaluation of machine learning (ML) models for each of the five follow-up endpoints.

	Sensitivity (%)	Specificity (%)	Accuracy (%)	AUC [95% CI]
**One-year mortality**				
Random forest	70.80	69.85	69.88	0.78 [0.74–0.82]
Naïve Bayes	59.85	85.67	84.96	0.78 [0.74–0.83]
SVM	63.50	74.65	74.35	0.74 [0.69–0.79]
XGBoost	66.42	77.73	77.42	0.79 [0.75–0.83]
KNN	68.61	63.52	63.66	0.72 [0.67–0.76]
Logistic regression	72.99	77.79	77.66	0.81 [0.77–0.85]
**Two-years mortality**				
Random forest	66.17	73.01	72.73	0.77 [0.73–0.80]
Naïve Bayes	76.62	62.39	62.98	0.76 [0.72–0.79]
SVM	48.76	85.26	83.73	0.67 [0.63–0.72]
XGBoost	66.17	72.62	72.35	0.75 [0.71–0.79]
KNN	63.18	69.50	69.24	0.73 [0.70–0.77]
Logistic regression	75.12	70.37	70.57	0.79 [0.76–0.82]
**Three-years mortality**				
Random forest	61.45	75.96	75.06	0.74 [0.71–0.77]
Naïve Bayes	58.55	80.03	78.70	0.74 [0.71–0.78]
SVM	45.09	84.53	82.08	0.68 [0.64–0.71]
XGBoost	68.00	67.11	67.16	0.74 [0.71–0.77]
KNN	70.18	64.92	65.24	0.72 [0.69–0.76]
Logistic regression	67.64	72.79	72.47	0.76 [0.73–0.79]
**Four-years mortality**				
Random forest	50.14	81.75	78.91	0.72 [0.69–0.75]
Naïve Bayes	53.03	81.44	78.88	0.71 [0.68–0.75]
SVM	42.07	83.32	79.61	0.64 [0.60–0.67]
XGBoost	65.71	65.84	65.83	0.70 [0.67–0.74]
KNN	67.72	63.70	64.06	0.70 [0.66–0.72]
Logistic regression	69.74	67.52	67.72	0.73 [0.70–0.76]
**Five-years mortality**				
Random forest	64.65	73.65	72.41	0.75 [0.72–0.77]
Naïve Bayes	58.14	77.67	74.98	0.73 [0.71–0.76]
SVM	48.37	80.61	76.16	0.66 [0.63–0.69]
XGBoost	65.35	72.98	71.93	0.74 [0.72–0.77]
KNN	60.70	73.20	71.48	0.73 [0.70–0.75]
Logistic regression	67.21	70.08	69.68	0.75 [0.72–0.77]

AUC, area under the receiver operating characteristic curve; CI, confidence interval; SVM, support vector machine; XGBoost, extreme gradient boosting; KNN: K-nearest neighbors.

**FIGURE 4:**
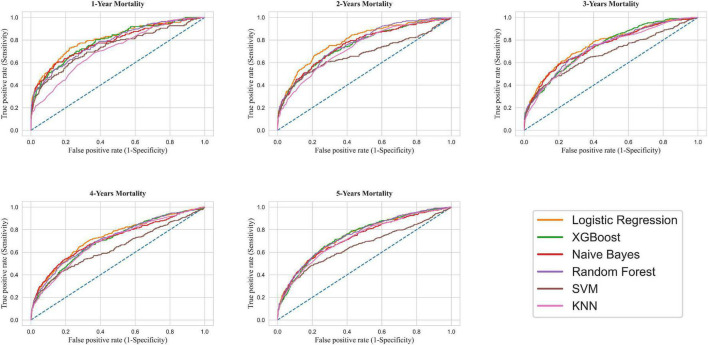
Receiver operating characteristic curve for mortality prediction. XGBoost, Extreme Gradient Boosting; SVM, Support Vector Machine; KNN, K-Nearest Neighbors.

By the same token, the LR model surpassed other models for predicting two-to-five-year mortality. Figure 4 demonstrates the ROC-AUC for all six models.

## Discussion

This study compared six ML models concerning one-to-five-year mortality among CABG patients. Our study revealed that the ventilation time after the surgery and baseline EF were by far the most influential factors for predicting mortality. Furthermore, the LR model had the best predictive ability for one-year mortality with excellent discrimination (AUC = 0.81) (10). Moreover, according to AUC interpretation, all ML models other than LR presented an acceptable performance for predicting one-year mortality (0.7 < AUC < 0.8) (10). The same performance trend was ascertained for two-to-five-year follow-ups.

CABG is one of the most prevalent surgeries worldwide. Several calculators and models have been developed to detect and minimize its main culprits of mortality and morbidity. As the era of big data analytics unfolds, ML algorithms are primed to have a considerable effect and improve contemporary risk calculators and scoring systems. Likewise, the need to have individualized systems to predict outcomes in operation-specific cohorts has highlighted the importance of ML models in recent years (12, 13). Methodologically, ML models allow us to adjust sensitivity and specificity in each clinical setting (14). Several techniques can be used in cases where we face a meager outcome, such as mortality, with the oversampling method being applied frequently. SMOTE oversampling creates synthetic examples for the minority group and is suggested to be better than undersampling methods because of retaining valuable data (15, 16). Lowering the prediction threshold is another common measure to overcome imbalanced data. As the 50% default threshold gives us many missed cases for mortality, we tuned this measure to have the optimum sensitivity and specificity on the ROC curve. This method has been used in several studies (14, 17).

Current risk scores such as STS, EURO-Score I and II were designed to predict short-term mortality and need to be modified to be used for long-term (2–4) as Puskas et al. (18) have proposed. Several models have been developed to predict the prognosis of CABG, with growing attention to ML methods. ML can be a promising tool for improving conventional scoring systems like STS (19, 20). Studies have reported the effectiveness of an ensemble of various ML algorithms concerning in-hospital mortality risk (21). A study investigated five ML methods to predict long-term mortality after CABG. In contrast to our study, this study concluded that Gradient Boosting Machine was the best predictive technique (AUC: 0.767), outperforming the LR technique (22). In agreement with this finding, another study compared various ML models for estimating the long-term mortality risk of the elderly who underwent CABG. Models included LR, RF, Classification And Regression Tree (CART), Multivariate Adaptive Regression Splines (MARS), and XGBoost. Their results showed that the XGBoost model and MARS had the best prediction performance, before and after variable selection, respectively (23).

However, like our findings, a recent meta-analysis concluded that in studies with low risk of bias, LR was as predictive as other ML models, while in studies at high risk of bias, other ML methods had better discrimination ability than LR (24). Despite these controversies, LR is a tried-and-true statistical method. It, therefore, should remain the gold standard until newer approaches can show demonstrably better predictive ability.

Many feature selection methods are available to select the most relevant features in ML models, with RF technique being commonly used in classification models (25). RF models work by constructing several random decision trees with the top features. For the classification, they use major vote among all decision trees to predict the class of the outcome (26).

Total ventilation hours as a peri-operative variable was the most important feature based on our feature selector. The importance of mechanical ventilation time for CABG mortality has also been supported by other studies using LR, RF, and XGBoost modeling (23). Other researchers have also indicated that mechanical ventilation requirement is a predictor of in-hospital and long-term mortality in patients undergoing cardiac surgery (27). In our study, EF was the second most important mortality predictor, followed by TG, age, Hb, Cr, total cholesterol, FBS, LDL-C, HDL-C, and BMI. The importance of these features has been reported in studies using ML methods or otherwise. These factors chime in with a recent study revealing creatinine, EF, and age among the most predictive features for in-hospital and 30-day mortality of cardiac surgery, using the XGBoost model (20). Similarly, a recent study on long-term survival of elderly patients with CABG revealed that age, renal disease, and hyperlipidemia are among the most important survival predictors, using various ML methods (23). The importance of EF on CABG survival has also been repeatedly reported in other studies (28, 29), some of which revealed a dose-response relationship between decreasing EF and overall risk of death (28). This is also the case with the role of our other selected features in estimating long-term CABG survival, including age (30, 31), glucose and lipid profile (32–35), Hb (36, 37), Cr (38, 39), and BMI (31).

Our cohort study was based on the cardiac surgery databank of Tehran Heart Center, one of Iran’s largest observational cardiovascular databases (40). The benefits of using this databank are as follows: (1) it included various demographic, preoperative, intraoperative, and postoperative information; (2) more than 16,000 and 10,000 patients could be tracked for a 1 and 5-year follow-up, respectively; (3) it could present current real-world experiences with CABG patients.

Nevertheless, there were some limitations to our study. As the study was conducted based on single-center data, the generalizability of models is a significant issue since the demographic characteristics of patients are confined to a single center (e.g., male predominance). Some relevant input features were discarded due to high missing data. There is also the potential for confounding variables that were not included in the analysis. No electrocardiogram data and follow-up laboratory results were available.

## Conclusion

**FIGURE 5:**
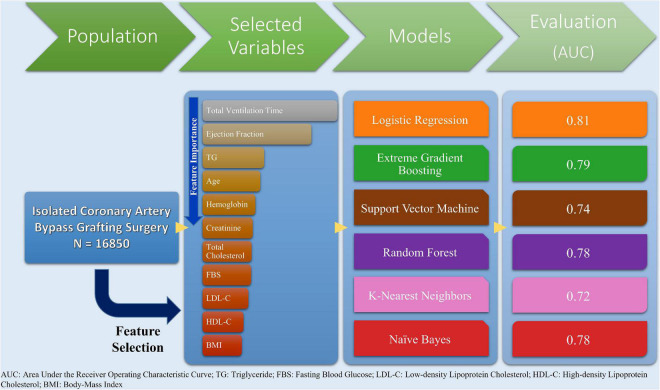
Summary of study design and machine learning models development and evaluation.

In this study, we developed different ML models for predicting one-to-five-year mortality in patients undergoing CABG. Feature selection chose eleven features for the prediction, the most vital of which were total ventilation hours and EF. Furthermore, all models demonstrated at least acceptable performance for estimating one-year mortality, with LR demonstrating the highest AUC (0.81). The overall summary of ML models and findings of our study is illustrated in Figure 5. In conclusion, ML algorithms may pave the way for clinicians to select CABG candidates through weighing mortality risks against the merits of receiving the surgery.

## Data availability statement

The raw data supporting the conclusions of this article will be made available by the authors, without undue reservation.

## Ethics statement

The studies involving human participants were reviewed and approved by the Ethics Committee of Tehran Heart Center, Tehran University of Medical Sciences, Tehran, Iran (IR.TUMS.VCR.REC.1400.11.23). The patients/participants provided their written informed consent to participate in this study.

## Author contributions

AK, AHB, KH, SS, JB, and SA contributed to the conception or design of the work. MP, MJ, AShe, and AF contributed to data acquisition. MP, AK, ASha, and AHB conducted data analysis. All authors contributed to data interpretation, participated in drafting the work or revising it critically for important intellectual content, approved the version for publication, and agreed to be accountable for all aspects of the work in ensuring that questions related to the accuracy or integrity of any part of the work are appropriately investigated and resolved.
